# *Bartonella quintana* and Typhus Group Rickettsiae Exposure among Homeless Persons, Bogotá, Colombia

**DOI:** 10.3201/eid2311.170341

**Published:** 2017-11

**Authors:** Álvaro A. Faccini-Martínez, Andrea C. Márquez, Diana M. Bravo-Estupiñan, Omar-Javier Calixto, Christian A. López-Castillo, Carlos A. Botero-García, Marylin Hidalgo, Claudia Cuervo

**Affiliations:** Universidade Federal do Espírito Santo, Vitória, Espírito Santo, Brazil (Á.A. Faccini-Martínez);; Pontificia Universidad Javeriana, Bogotá, Colombia (A.C. Márquez, D.M. Bravo-Estupiñan, M. Hidalgo, C. Cuervo);; Hasselt University, Brussels, Belgium (O.-J. Calixto);; Asociación Colombiana de Infectología, Bogotá (C.A. López-Castillo);; Hospital Militar Central, Bogotá (C.A. Botero-García)

**Keywords:** Bartonella quintana, Rickettsia, homeless persons, Colombia, lice, vector-borne infections, Brazil, arthropods, bacteria, typhus group rickettsiae

## Abstract

In 2015, we investigated *Bartonella quintana* and typhus group rickettsiae in body lice from homeless persons in Bogotá, Colombia. We found *B. quintana*–infected body lice and seroprevalence of this microorganism in 19% of homeless persons and typhus group rickettsiae in 56%. Public health professionals should start preemptive measures and active vector control.

Homeless persons make up part of the population at highest risk for infectious diseases because of factors such as deficient hygiene habits, infrequent washing and changing clothes, and overcrowding (*1*). Within this group, vector-borne diseases caused by bacteria of the genera *Bartonella*, *Rickettsia*, and *Borrelia* are of great importance; louseborne *B. quintana* is the main microorganism associated with infections in homeless persons (*1*). In Colombia, the presence of *R. prowazekii*, a typhus group rickettsiae (TGR), in lice and related human infections in Bogotá was evident only during 1918–1922 and 1941 (*2*). We investigated the presence of *B. quintana* and TGR in body lice collected from homeless persons in Bogotá and these persons’ exposure to such microorganisms.

## The Study

The Research and Ethics Committees of the Facultad de Ciencias of the Pontificia Universidad Javeriana (Bogotá, Colombia) approved this study (February 14, 2013). All participants read, accepted, and signed the informed consent form.

**Figure 1:**
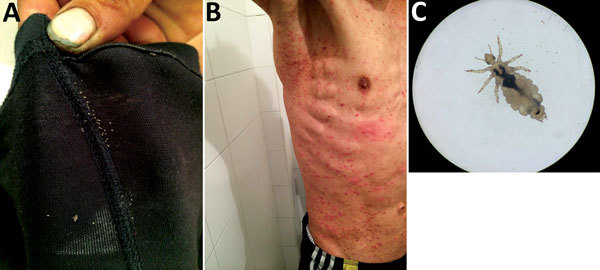
Homeless man infested by body lice, Bogotá, Colombia, 2015. A) Body lice and eggs in clothing seams. B) Pruritic and scratching lesions on the man’s body. C) Adult female body louse collected from clothing.

During May–September 2015, we enrolled a total of 153 persons from a homeless shelter in Bogotá in the study and obtained serum samples from each person. We also collected lice from participants’ clothing or body (below the neck) and considered these lice a positive indication of body louse infestation. Of the study participants, 132 were men, 17 were women, and 4 were transgender (for the study, people who had a gender identity or gender expression that differs from their assigned sex). Participants’ mean age was 39.6 (SD ± 11.65) years. Eighteen (11.7%) were infested with body lice; lice were preliminarily identified as *Pediculus humanus humanus*, according to standard taxonomic keys (*3*), and all had the same light color (Figure 1).

To detect specific IgG to *Bartonella* spp. (*B. quintana* antigen) and TGR (*R. typhi* antigen) in serum samples, we used commercially sourced indirect fluorescent antibody (IFA) kits (Bartonella IFA IgG and Rickettsia IFA IgG; Focus Technologies, Cypress, CA, USA). We screened serum at a dilution of 1:64. Among the 153 study participants, 29 (19.0%) had IgG that reacted exclusively against *Bartonella* spp. at a titer of >64, and 86 (56.2%) had IgG that reacted exclusively against TGR at a titer of >64. Twenty (13.1%) participants had IgG against both *Bartonella* spp. and TGR.

**Figure 2:**
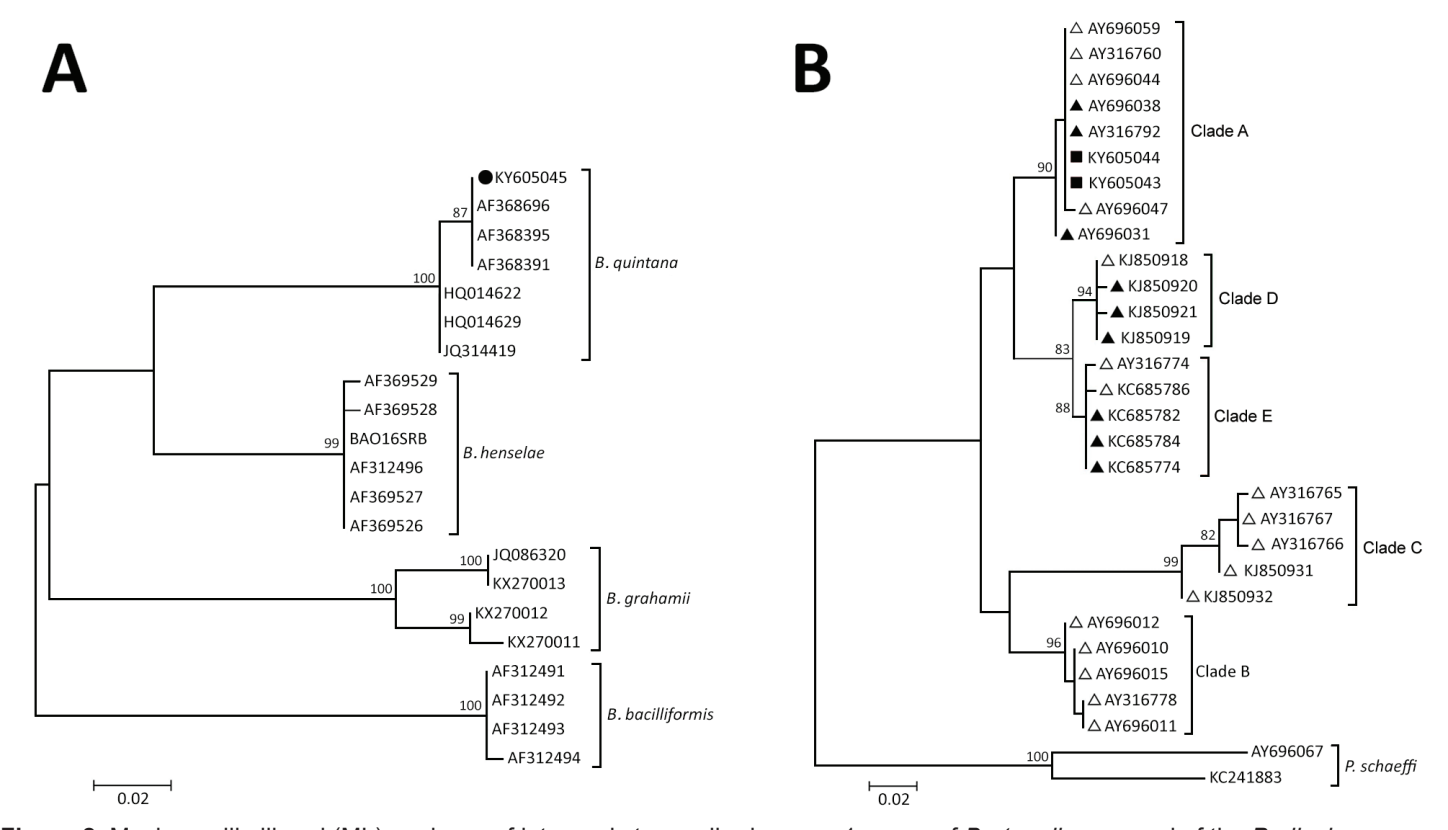
Maximum-likelihood (ML) analyses of intergenic transcribed spacer 1 genes of *Bartonella* spp. and of the *Pediculus humanus* *humanus* louse mitochondrial cytochrome b (*cytb*) gene. A) *Bartonella* spp. analysis. The tree with the highest log likelihood is shown, and ML bootstrap values >80 are indicated at each node. The tree is drawn to scale. Sequences are indicated by GenBank accession number; solid circle indicates the sequence retrieved in this study. The *Bartonella* species is indicated to the right of each branch. B) *P. h.* *humanus* analysis. The tree with the highest log likelihood is shown, and ML bootstrap values are located above the node. The tree is drawn to scale. The body louse sequences (solid triangles), head louse sequences (open triangles), and GenBank accession numbers are indicated. Cytochrome b sequences from *Pediculus schaeffi* were used as outgroups. Solid squares indicate the sequences retrieved in this study. The mitochondrial clade is indicated to the right of each branch. Scale bars indicate nucleotide substitutions per site.

For identification of *Bartonella* spp. and *Rickettsia* spp. from louse samples, we organized 201 body lice into 39 pools and extracted DNA from each pool (DNeasy Blood and Tissue; QIAGEN, Valencia, CA, USA). We screened all louse pools by standard PCR for *Bartonella* spp. (citrate synthase gene [*gltA*] and 16S–23S rRNA intergenic transcribed spacer region [ITS-1]) and *Rickettsia* spp. (*gltA*, 16S RNA, and *ompB* rickettsial genes) as described (*4*). Eleven (28%) louse pools were positive for *Bartonella* spp., of which 7 were positive for *gltA*, 10 were positive for ITS-1, and 6 were positive for both genes. We found no evidence of *Rickettsia* spp. infection in body lice. For *Bartonella* spp., sequence of the ITS-1 fragment amplified by standard PCR and phylogenetic analysis with maximum-likelihood method and 1,000 bootstrap replicates, performed using MEGA software version 6 (*5*), confirmed the bacteria as *B. quintana* (GenBank accession no. KY605045) (Figure 2, panel A).

To examine the mitochondrial clade in *P. h. humanus* captured in this study, we tested DNA samples from 2 randomly selected lice and PCR amplified the *cytb* genes as reported (*6*). We compared the *cytb* sequences obtained in our study with known head and body louse sequences from the 5 *P. h. humanus* clades (A–E) (*6*). Phylogenetic analysis using the maximum-likelihood method demonstrated that the 2 sequences obtained in our study belong to clade A (GenBank accession nos. KY605043 and KY605044) (Figure 2, panel B).

## Conclusions

Our study demonstrates evidence of infestation by the body louse (*P. h. humanus*) infected with *B. quintana* and exposure to TGR in homeless persons in Bogotá. The rate of body louse infestation in the studied population (11.7%) was within the range reported elsewhere (7%–30%) (*1*,*7*), confirming that homeless persons are among the population groups most vulnerable to parasitism by this arthropod and associated infectious agents (*1*). We also found seroprevalence for *Bartonella* spp. (19.0%) in line with the range reported in other studies worldwide (0.4%–62%) (*8*,*9*). Although cross-reactions in the IFA between different *Bartonella* species are possible (highlighting those associated with homeless persons: *B. quintana*, *B. elizabethae*, and *B. henselae*) (*8*,*9*), we consider that the seroprevalence detected in homeless persons in Bogotá probably is due to *B. quintana* because it is the microorganism most frequently associated with homeless persons (*1*), and we detected it in 28.2% of body lice collected from persons sampled, again in agreement with previous studies (1.4%–94%) (*10*).

On the other hand, the level of seropositivity against TGR found in our study (56.2%) was considerably higher than levels reported in previous studies (≈0.54%–22%) (*9*). We were not able to perform a Western blot–associated cross-adsorption test to distinguish the specific *Rickettsia* species involved in the TGR-positive serum (*11*). However, we consider that *R. typhi* was probably the predominant species responsible for the seropositivity for the following reasons: *R. prowazekii* was not detected in collected body lice; infection with *R. typhi* is frequent in homeless persons (*11*); and no records from healthcare government entities in Colombia, whether local (Bogotá) or national, suggest the occurrence in this population of febrile illness with high death rates, which would be more compatible with the epidemiology of epidemic typhus (*R. prowazekii* infection) than with murine typhus (*R. typhi* infection) (*12*). Although human body lice are not clearly identified vectors of *R. typhi*, it seems that under certain circumstances they could transmit *R. typhi* (*13*), as well as other rickettsiae (*14*). More work is needed to identify properly the *Rickettsia* and *Bartonella* species involved in this antibody prevalence.

We identified the lice collected from homeless persons in Bogotá as belonging to the haplogroup/clade A, which is distributed worldwide and comprises *P. h. humanus* and *P. h. capitis* (*6*). In our study, given that lice were collected from clothes and body areas below the neck, they probably were *P. h. humanus*. Nevertheless, molecular determination by PCR using the Phum_PHUM540560 gene currently is the only way to distinguish body and head lice (*15*).

Our study is subject to several limitations. The detection of *Bartonella* spp. and *Rickettsia* spp. was based on standard PCR and not on real-time PCR, which is more sensitive. As a result, some samples could have tested negative because of low DNA load. Moreover, we did not test serum or blood samples by molecular assays, nor perform cultures to isolate infectious agents from the lice. Nonetheless, our study results should encourage public health professionals in Bogotá to start preemptive measures and active vector control (delousing and ivermectim treatment) (*1*), conduct future research evaluating the clinical characteristics of *Bartonella* and *Rickettsia* infections in homeless persons in Bogotá, confirm circulation of specific species of these microorganisms, and screen for *Bartonella* spp. endocarditis by blood culture among homeless persons who have high antibody titers.
